# A mobile app for tracking non-motor symptoms of people with Parkinson’s disease: A usability study

**DOI:** 10.1093/geroni/igaa057.3284

**Published:** 2020-12-16

**Authors:** JuHee Lee, Yujin Suh, Yielin Kim

**Affiliations:** Yonsei University, Seoul, Republic of Korea

## Abstract

Smart phone-based technology for people with Parkinson’s disease has been developed worldwide. Unmonitored non-motor symptoms decrease quality of life of people with Parkinson’s disease, so the needs for technology to manage non-motor symptoms are increasing. The technology is needed to detect subtle changes in non-motor symptoms by healthcare professional. There is no mobile app which manage comprehensive symptoms of Parkinson’s disease including non-motor symptoms. It is necessary to develop a new tracking system that can effectively manage non-motor symptoms as well as motor symptoms of Parkinson’s disease. We developed a prototype of mobile app for Android smartphones, with cooperation with Mazelone company. we also have shaped functions for monitoring of motor symptoms and medication adherence. It also provided a section for caregivers to use on behalf of people with Parkinson’s disease who have difficulty to use app due to hand tremor. Through Delphi technique, we obtained content validity from eight medical and nursing experts on the contents of the application. We provided regular telephone counseling to improve and encourage their app usage. Fifteen participants used the app for 6 weeks. To evaluate usability of mobile app, we provided constructed questionnaire and conducted individual telephone interview. A mobile app for tracking non-motor symptoms demonstrated high usability and satisfaction. We learned lessons about facilitators and barriers when implementing an app such as perception and acceptance of mobile technology. The mobile app will improve continuum of care. Future studies need to improve the contents and refine technical approach for people with Parkinson’s disease.

